# Personal

**Published:** 1899-03

**Authors:** 


### PERSONAL.

DR. GEORGE H. SIMMONS, of Lincoln, Neb., was chosen editor of the *Journal of the American Medical Association* at a regular meeting of the board of trustees, held at Chicago, February 17, 1899. Dr. Simmons has been editor of the *Western Medical Review* since it was established. He is a vigorous writer and a man of affairs, hence his work in his new field is likely to prove agreeable and successful.

Dr. George B. Stocker and Dr. B. P. Hoyer, of Buffalo, were appointed post mortem examiners for the County of Erie by the board of supervisors at the annual meeting, held in January. In the City of New York the coroner's physician is the official designation given to analogous service.

Dr. G. A. Himmelsbach, of Buffalo, has gone to St. Augustine, Fla., to enjoy the balmy atmosphere of that delightful region. He was accompanied by Mrs. Himmelsbach and they sailed from New York by the Clyde Line early in February.

Dr. N. W. Aldrich, of Buffalo, has been appointed junior assistant physician at the Buffalo State Hospital.

Dr. W. H. Mansperger, of Buffalo, has removed from 66 West Chippewa street to 1003 Main street.

Dr. H. C. Rooth, of Buffalo, was recently appointed a member of the Riverside Hospital staff.

